# Crystal structure of 2,2-dimethyl-*N*-(pyridin-3-yl)propanamide

**DOI:** 10.1107/S2056989015005289

**Published:** 2015-03-21

**Authors:** Gamal A. El-Hiti, Keith Smith, Amany S. Hegazy, Saud A. Alanazi, Benson M. Kariuki

**Affiliations:** a Cornea Research Chair, Department of Optometry, College of Applied Medical Sciences, King Saud University, PO Box 10219, Riyadh 11433, Saudi Arabia; b School of Chemistry, Cardiff University, Main Building, Park Place, Cardiff CF10 3AT, Wales

**Keywords:** crystal structure, pyridine, propanamide, N—H⋯N hydrogen bonds

## Abstract

In the title compound, C_10_H_14_N_2_O, the pyridine ring is inclined to the mean plane of the amide moiety [N—C(=O)C] by 17.60 (8)°. There is an intra­molecular C—H⋯O hydrogen bond present involving the carbonyl O atom. In the crystal, mol­ecules are linked *via* N—H⋯N hydrogen bonds, forming chains propagating along [100]. The *tert*-butyl group is disordered over two sets of sites with a refined occupancy ratio of 0.758 (12):0.242 (12).

## Related literature   

For related biologically active pyridine derivatives, see: de Candia *et al.* (2013 ▸); Thorat *et al.* (2013 ▸); Abdel-Megeed *et al.* (2012 ▸). For pyridine ring-system modifications, see: El-Hiti *et al.* (2015 ▸); Smith *et al.* (2012 ▸, 2013 ▸); Londregan *et al.* (2009 ▸); Joule & Mills (2000 ▸); Turner (1983 ▸). For the crystal structures of related compounds, see: El-Hiti *et al.* (2014 ▸); Seidler *et al.* (2011 ▸); Koch *et al.* (2008 ▸); Mazik *et al.* (2004 ▸).
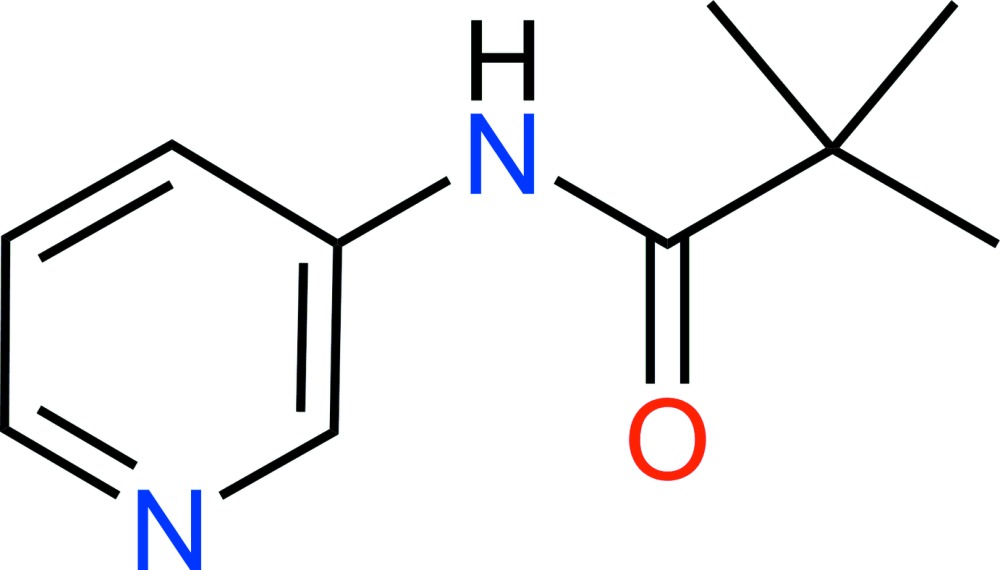


## Experimental   

### Crystal data  

C_10_H_14_N_2_O
*M* *_r_* = 178.23
Orthorhombic, 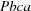

*a* = 11.2453 (3) Å
*b* = 10.5272 (3) Å
*c* = 17.5339 (6) Å
*V* = 2075.69 (11) Å^3^
*Z* = 8
Cu *K*α radiation
μ = 0.60 mm^−1^
*T* = 293 K
0.23 × 0.19 × 0.06 mm

### Data collection  

Agilent SuperNova (Dual, Cu at zero, Atlas) diffractometer
Absorption correction: multi-scan (*CrysAlis PRO*; Agilent, 2014 ▸) *T*_min_ = 0.840, *T*_max_ = 1.000
7164 measured reflections
2065 independent reflections
1722 reflections with *I* > 2σ(*I*)
*R*_int_ = 0.017

### Refinement  

*R*[*F* ^2^ > 2σ(*F* ^2^)] = 0.041
*wR*(*F* ^2^) = 0.127
*S* = 1.05
2065 reflections
153 parameters
114 restraints
H-atom parameters constrained
Δρ_max_ = 0.19 e Å^−3^
Δρ_min_ = −0.16 e Å^−3^

### 

Data collection: *CrysAlis PRO* (Agilent, 2014 ▸); cell refinement: *CrysAlis PRO*; data reduction: *CrysAlis PRO*; program(s) used to solve structure: *SHELXS2013* (Sheldrick, 2008 ▸); program(s) used to refine structure: *SHELXL2013* (Sheldrick, 2015 ▸); molecular graphics: *ORTEP-3 for Windows* (Farrugia, 2012 ▸); software used to prepare material for publication: *WinGX* (Farrugia, 2012 ▸).

## Supplementary Material

Crystal structure: contains datablock(s) I, New_Global_Publ_Block. DOI: 10.1107/S2056989015005289/su5094sup1.cif

Structure factors: contains datablock(s) I. DOI: 10.1107/S2056989015005289/su5094Isup2.hkl

**Fig. 1.**
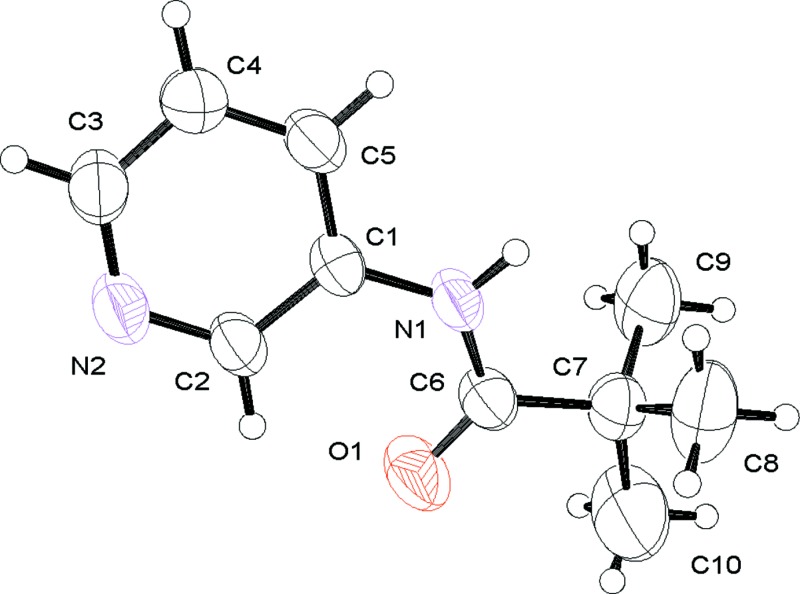
The molecular structure of the title compound, with atom labelling. Displacement ellipsoids are drawn at the 50% probability level. Only the major component of the disordered t-butyl group is shown.

**Fig. 2.**
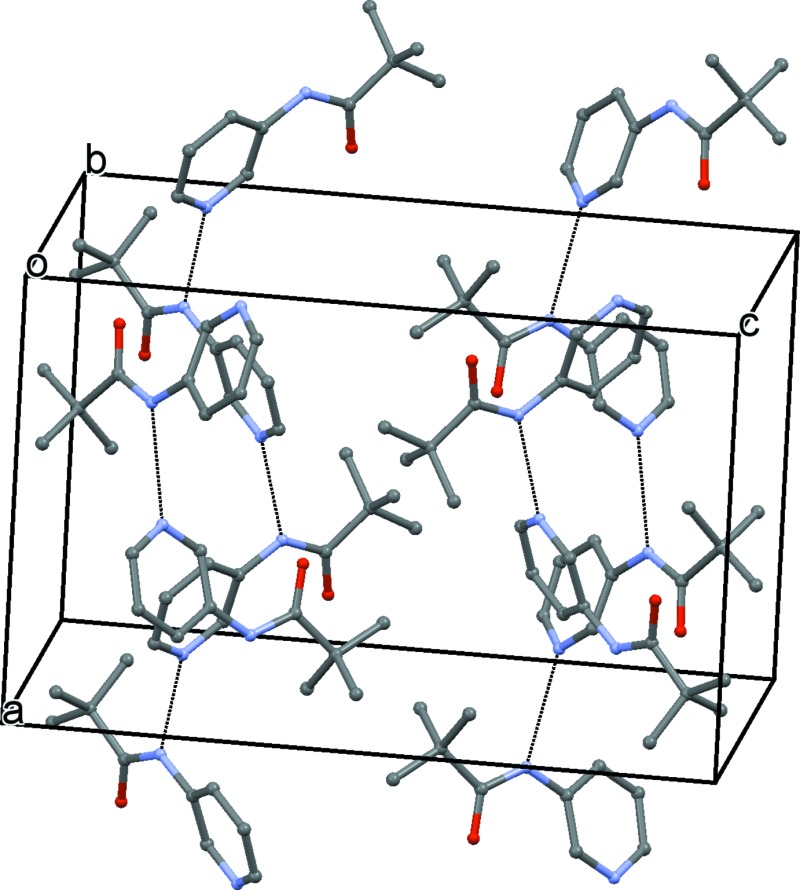
Crystal packing of the title compound, viewed along the b axis, with the N—H···N interactions shown as dashed lines (see Table 1 for details). The minor component of the disordered t-butyl group has been omitted for clarity.

Supporting information file. DOI: 10.1107/S2056989015005289/su5094Isup3.cml

t . DOI: 10.1107/S2056989015005289/su5094fig1.tif
The mol­ecular structure of the title compound, with atom labelling. Displacement ellipsoids are drawn at the 50% probability level. Only the major component of the disordered *t*-butyl group is shown.

b t . DOI: 10.1107/S2056989015005289/su5094fig2.tif
Crystal packing of the title compound, viewed along the *b* axis, with the N—H⋯N inter­actions shown as dashed lines (see Table 1 for details). The minor component of the disordered *t*-butyl group has been omitted for clarity.

CCDC reference: 1054113

Additional supporting information:  crystallographic information; 3D view; checkCIF report

## Figures and Tables

**Table 1:** Hydrogen-bond geometry (, )

*D*H*A*	*D*H	H*A*	*D* *A*	*D*H*A*
C2H2O1	0.93	2.25	2.8263(18)	119
N1H1N2^i^	0.86	2.17	3.0012(15)	164

Symmetry code: (i) 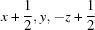.

## supplementary crystallographic information

### S1. Introduction

Pyridine derivatives are inter­esting compounds (Joule & Mills, 2000) since they show a range of biological activities (Thorat *et al.*, 2013) such as anti­coagulant (de Candia *et al.*, 2013) and anti­microbial (Abdel-Megeed *et al.*, 2012) properties. Various simple and efficient processes have been developed for modification of the pyridine ring system (El-Hiti *et al.*, 2015; Smith *et al.*, 2013, Smith *et al.*, 2012, Londregan *et al.*, 2009; Turner, 1983). The X-ray crystal structures of related compounds have been reported (El-Hiti *et al.*, 2014; Seidler *et al.*, 2011; Koch *et al.*, 2008; Mazik *et al.*, 2004).

### S2. Experimental

The title compound was obtained in 73% yield from the reaction of 3-amino­pyridine with pivaloyl chloride in the presence of tri­ethyl­amine in di­chloro­methane at 273 K for 15 min and then at room temperature for 2 h (Turner, 1983). Crystallization from a mixture of ethyl acetate and hexane gave colourless crystals of the title compound. The spectroscopic and analytical data for the title compound were identical with those reported previously (Turner, 1983)

### S2.1. Refinement

The N- and C-bound H atoms were included in calculated positions and refined as riding: N—H = 0.86 Å, C—H = 0.93 - 0.98 Å with U_iso_(H) = 1.5U_eq_(C) for methyl H atoms and = 1.2U_eq_(C) for other H atoms. The *t*-butyl group is disordered over two sites and was refined with bond length constraints to give a refined occupancy ratio of 0.758 (12):0.242 (12).

### S3. Results and discussion

The molecular structure of the title compound is illustrated in Fig. 1. The pyridine ring is inclined to the mean plane of the amide moiety [N1—C6(═ O1)—C7] by 17.60 (8) °. There is an intra­molecular C—H···O hydrogen bond present involving the carbonyl O atom (Table 1).

In the crystal, molecules are linked via N—H···N hydrogen bonds forming chains propagating along [100]; see Table 1 and Fig. 2.

### Crystal data

**Table:**

C_10_H_14_N_2_O	*D*_x_ = 1.141 Mg m^−^^3^
*M**_r_* = 178.23	Cu *K*α radiation, λ = 1.54184 Å
Orthorhombic, *P**b**c**a*	Cell parameters from 3061 reflections
*a* = 11.2453 (3) Å	θ = 5.0–73.4°
*b* = 10.5272 (3) Å	µ = 0.60 mm^−^^1^
*c* = 17.5339 (6) Å	*T* = 293 K
*V* = 2075.69 (11) Å^3^	Plate, colourless
*Z* = 8	0.23 × 0.19 × 0.06 mm
*F*(000) = 768	

### Data collection

**Table:**

Agilent SuperNova (Dual, Cu at zero, Atlas) diffractometer	2065 independent reflections
Radiation source: sealed X-ray tube, SuperNova (Cu) X-ray Source	1722 reflections with *I* > 2σ(*I*)
Mirror monochromator	*R*_int_ = 0.017
ω scans	θ_max_ = 73.8°, θ_min_ = 5.1°
Absorption correction: multi-scan (*CrysAlis PRO*; Agilent, 2014)	*h* = −14→8
*T*_min_ = 0.840, *T*_max_ = 1.000	*k* = −13→12
7164 measured reflections	*l* = −20→21

### Refinement

**Table:**

Refinement on *F*^2^	Hydrogen site location: inferred from neighbouring sites
Least-squares matrix: full	H-atom parameters constrained
*R*[*F*^2^ > 2σ(*F*^2^)] = 0.041	*w* = 1/[σ^2^(*F*_o_^2^) + (0.0688*P*)^2^ + 0.2387*P*] where *P* = (*F*_o_^2^ + 2*F*_c_^2^)/3
*wR*(*F*^2^) = 0.127	(Δ/σ)_max_ < 0.001
*S* = 1.05	Δρ_max_ = 0.19 e Å^−^^3^
2065 reflections	Δρ_min_ = −0.16 e Å^−^^3^
153 parameters	Extinction correction: *SHELXL2013* (Sheldrick, 2015), Fc^*^=kFc[1+0.001xFc^2^λ^3^/sin(2θ)]^-1/4^
114 restraints	Extinction coefficient: 0.0016 (4)

### Special details

**Table:**

Geometry. All e.s.d.'s (except the e.s.d. in the dihedral angle between two l.s. planes) are estimated using the full covariance matrix. The cell e.s.d.'s are taken into account individually in the estimation of e.s.d.'s in distances, angles and torsion angles; correlations between e.s.d.'s in cell parameters are only used when they are defined by crystal symmetry. An approximate (isotropic) treatment of cell e.s.d.'s is used for estimating e.s.d.'s involving l.s. planes.

### Fractional atomic coordinates and isotropic or equivalent isotropic displacement parameters (Å^2^)

**Table:**

	*x*	*y*	*z*	*U*_iso_*/*U*_eq_	Occ. (<1)
C1	0.72804 (10)	0.10403 (12)	0.27358 (7)	0.0490 (3)	
C2	0.61380 (11)	0.14128 (14)	0.25328 (8)	0.0585 (4)	
H2	0.5822	0.2140	0.2756	0.070*	
C3	0.59204 (13)	−0.02662 (15)	0.17123 (8)	0.0663 (4)	
H3	0.5452	−0.0722	0.1371	0.080*	
C4	0.70473 (13)	−0.06915 (16)	0.18680 (9)	0.0702 (4)	
H4	0.7344	−0.1414	0.1630	0.084*	
C5	0.77323 (12)	−0.00301 (14)	0.23832 (8)	0.0619 (4)	
H5	0.8499	−0.0303	0.2494	0.074*	
C6	0.76123 (12)	0.24951 (14)	0.38234 (8)	0.0585 (3)	
C7	0.85629 (13)	0.29549 (15)	0.43884 (9)	0.0671 (4)	
C8	0.9594 (3)	0.3605 (4)	0.3935 (2)	0.0798 (10)	0.758 (12)
H8A	0.9277	0.4278	0.3627	0.120*	0.758 (12)
H8B	0.9974	0.2988	0.3613	0.120*	0.758 (12)
H8C	1.0165	0.3947	0.4287	0.120*	0.758 (12)
C9	0.9077 (5)	0.1835 (4)	0.4813 (4)	0.0838 (11)	0.758 (12)
H9A	0.9680	0.2125	0.5159	0.126*	0.758 (12)
H9B	0.9421	0.1250	0.4455	0.126*	0.758 (12)
H9C	0.8458	0.1417	0.5094	0.126*	0.758 (12)
C10	0.8023 (4)	0.3941 (7)	0.4916 (4)	0.1205 (18)	0.758 (12)
H10A	0.7365	0.3574	0.5186	0.181*	0.758 (12)
H10B	0.7751	0.4653	0.4621	0.181*	0.758 (12)
H10C	0.8613	0.4222	0.5274	0.181*	0.758 (12)
C8A	0.9208 (16)	0.4067 (14)	0.4095 (8)	0.108 (4)	0.242 (12)
H8D	0.9534	0.3873	0.3602	0.162*	0.242 (12)
H8E	0.9841	0.4281	0.4439	0.162*	0.242 (12)
H8F	0.8672	0.4772	0.4052	0.162*	0.242 (12)
C9A	0.9385 (16)	0.1880 (15)	0.4658 (11)	0.089 (4)	0.242 (12)
H9D	0.8920	0.1137	0.4766	0.133*	0.242 (12)
H9E	0.9796	0.2142	0.5111	0.133*	0.242 (12)
H9F	0.9953	0.1690	0.4265	0.133*	0.242 (12)
C10A	0.7832 (11)	0.3342 (17)	0.5131 (6)	0.098 (4)	0.242 (12)
H10D	0.7211	0.3924	0.4993	0.147*	0.242 (12)
H10E	0.8355	0.3739	0.5491	0.147*	0.242 (12)
H10F	0.7488	0.2595	0.5355	0.147*	0.242 (12)
N1	0.79882 (8)	0.16796 (11)	0.32735 (6)	0.0543 (3)	
H1	0.8741	0.1539	0.3252	0.065*	
N2	0.54740 (9)	0.07752 (13)	0.20320 (7)	0.0650 (3)	
O1	0.65818 (9)	0.28385 (14)	0.38687 (7)	0.0885 (4)	

### Atomic displacement parameters (Å^2^)

**Table:**

	*U*^11^	*U*^22^	*U*^33^	*U*^12^	*U*^13^	*U*^23^
C1	0.0369 (6)	0.0624 (7)	0.0478 (6)	0.0007 (5)	0.0006 (5)	0.0031 (5)
C2	0.0400 (6)	0.0715 (8)	0.0640 (7)	0.0064 (6)	−0.0046 (5)	−0.0063 (6)
C3	0.0529 (7)	0.0841 (9)	0.0617 (8)	−0.0037 (7)	−0.0065 (6)	−0.0111 (7)
C4	0.0630 (9)	0.0778 (9)	0.0697 (9)	0.0116 (7)	−0.0067 (7)	−0.0172 (7)
C5	0.0450 (7)	0.0766 (8)	0.0641 (8)	0.0130 (6)	−0.0051 (6)	−0.0078 (6)
C6	0.0461 (7)	0.0715 (8)	0.0579 (7)	0.0057 (6)	−0.0020 (6)	−0.0039 (6)
C7	0.0620 (8)	0.0739 (8)	0.0653 (8)	−0.0024 (7)	−0.0092 (6)	−0.0103 (7)
C8	0.0716 (17)	0.0753 (17)	0.0926 (19)	−0.0182 (13)	−0.0135 (13)	0.0017 (14)
C9	0.089 (3)	0.101 (2)	0.062 (2)	−0.0202 (16)	−0.0235 (18)	0.0146 (16)
C10	0.098 (2)	0.134 (4)	0.129 (4)	−0.002 (3)	−0.004 (2)	−0.073 (3)
C8A	0.129 (8)	0.107 (7)	0.087 (6)	−0.026 (6)	−0.027 (6)	0.000 (6)
C9A	0.080 (7)	0.116 (7)	0.071 (7)	0.008 (6)	−0.023 (5)	−0.011 (5)
C10A	0.102 (6)	0.115 (8)	0.076 (5)	0.013 (6)	−0.026 (4)	−0.046 (5)
N1	0.0341 (5)	0.0708 (7)	0.0581 (6)	0.0041 (4)	−0.0035 (4)	−0.0056 (5)
N2	0.0408 (6)	0.0867 (8)	0.0676 (7)	0.0032 (5)	−0.0078 (5)	−0.0079 (6)
O1	0.0543 (6)	0.1273 (10)	0.0838 (8)	0.0235 (6)	−0.0048 (5)	−0.0356 (7)

### Geometric parameters (Å, º)

**Table:**

C1—C5	1.3820 (19)	C8—H8A	0.9600
C1—C2	1.3895 (17)	C8—H8B	0.9600
C1—N1	1.4054 (16)	C8—H8C	0.9600
C2—N2	1.3339 (18)	C9—H9A	0.9600
C2—H2	0.9300	C9—H9B	0.9600
C3—N2	1.3297 (19)	C9—H9C	0.9600
C3—C4	1.372 (2)	C10—H10A	0.9600
C3—H3	0.9300	C10—H10B	0.9600
C4—C5	1.376 (2)	C10—H10C	0.9600
C4—H4	0.9300	C8A—H8D	0.9600
C5—H5	0.9300	C8A—H8E	0.9600
C6—O1	1.2165 (17)	C8A—H8F	0.9600
C6—N1	1.3585 (17)	C9A—H9D	0.9600
C6—C7	1.5356 (19)	C9A—H9E	0.9600
C7—C8A	1.470 (7)	C9A—H9F	0.9600
C7—C9	1.510 (4)	C10A—H10D	0.9600
C7—C10	1.517 (4)	C10A—H10E	0.9600
C7—C9A	1.536 (8)	C10A—H10F	0.9600
C7—C8	1.563 (3)	N1—H1	0.8600
C7—C10A	1.593 (7)		
			
C5—C1—C2	117.09 (12)	H8B—C8—H8C	109.5
C5—C1—N1	118.83 (10)	C7—C9—H9A	109.5
C2—C1—N1	124.07 (11)	C7—C9—H9B	109.5
N2—C2—C1	122.97 (12)	H9A—C9—H9B	109.5
N2—C2—H2	118.5	C7—C9—H9C	109.5
C1—C2—H2	118.5	H9A—C9—H9C	109.5
N2—C3—C4	122.28 (13)	H9B—C9—H9C	109.5
N2—C3—H3	118.9	C7—C10—H10A	109.5
C4—C3—H3	118.9	C7—C10—H10B	109.5
C3—C4—C5	118.86 (14)	H10A—C10—H10B	109.5
C3—C4—H4	120.6	C7—C10—H10C	109.5
C5—C4—H4	120.6	H10A—C10—H10C	109.5
C4—C5—C1	120.02 (12)	H10B—C10—H10C	109.5
C4—C5—H5	120.0	C7—C8A—H8D	109.5
C1—C5—H5	120.0	C7—C8A—H8E	109.5
O1—C6—N1	122.04 (13)	H8D—C8A—H8E	109.5
O1—C6—C7	121.83 (13)	C7—C8A—H8F	109.5
N1—C6—C7	116.13 (11)	H8D—C8A—H8F	109.5
C9—C7—C10	112.8 (3)	H8E—C8A—H8F	109.5
C8A—C7—C6	111.6 (5)	C7—C9A—H9D	109.5
C9—C7—C6	109.8 (3)	C7—C9A—H9E	109.5
C10—C7—C6	109.3 (2)	H9D—C9A—H9E	109.5
C8A—C7—C9A	113.4 (7)	C7—C9A—H9F	109.5
C6—C7—C9A	112.7 (9)	H9D—C9A—H9F	109.5
C9—C7—C8	107.9 (2)	H9E—C9A—H9F	109.5
C10—C7—C8	107.9 (2)	C7—C10A—H10D	109.5
C6—C7—C8	109.05 (17)	C7—C10A—H10E	109.5
C8A—C7—C10A	109.7 (5)	H10D—C10A—H10E	109.5
C6—C7—C10A	104.4 (5)	C7—C10A—H10F	109.5
C9A—C7—C10A	104.3 (6)	H10D—C10A—H10F	109.5
C7—C8—H8A	109.5	H10E—C10A—H10F	109.5
C7—C8—H8B	109.5	C6—N1—C1	127.05 (10)
H8A—C8—H8B	109.5	C6—N1—H1	116.5
C7—C8—H8C	109.5	C1—N1—H1	116.5
H8A—C8—H8C	109.5	C3—N2—C2	118.76 (11)

### Hydrogen-bond geometry (Å, º)

**Table:**

*D*—H···*A*	*D*—H	H···*A*	*D*···*A*	*D*—H···*A*
C2—H2···O1	0.93	2.25	2.8263 (18)	119
N1—H1···N2^i^	0.86	2.17	3.0012 (15)	164

Symmetry code: (i) *x*+1/2, *y*, −*z*+1/2.
