# Supplementary figures and images for: Crystal structure of 2,2-dimethyl-N-(pyridin-3-yl)propanamide
Source: Acta Crystallogr E Crystallogr Commun. 2015 Mar 21;71(Pt 4):o246–7. doi: 10.1107/S2056989015005289 (PMC4438820; doi:10.1107/S2056989015005289)

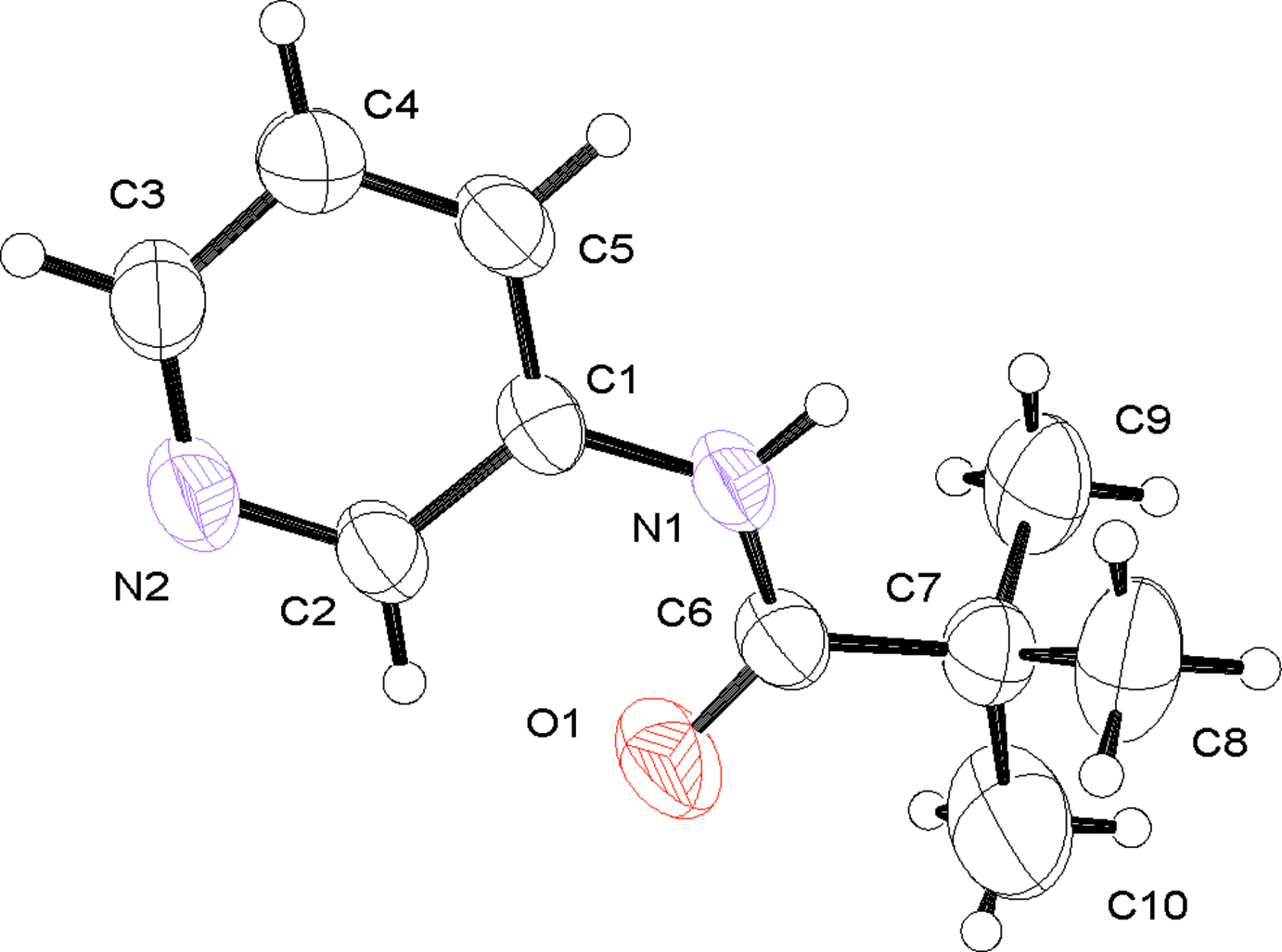

Supplement: Supplementary file 4 [file e-71-0o246-fig1.tif]

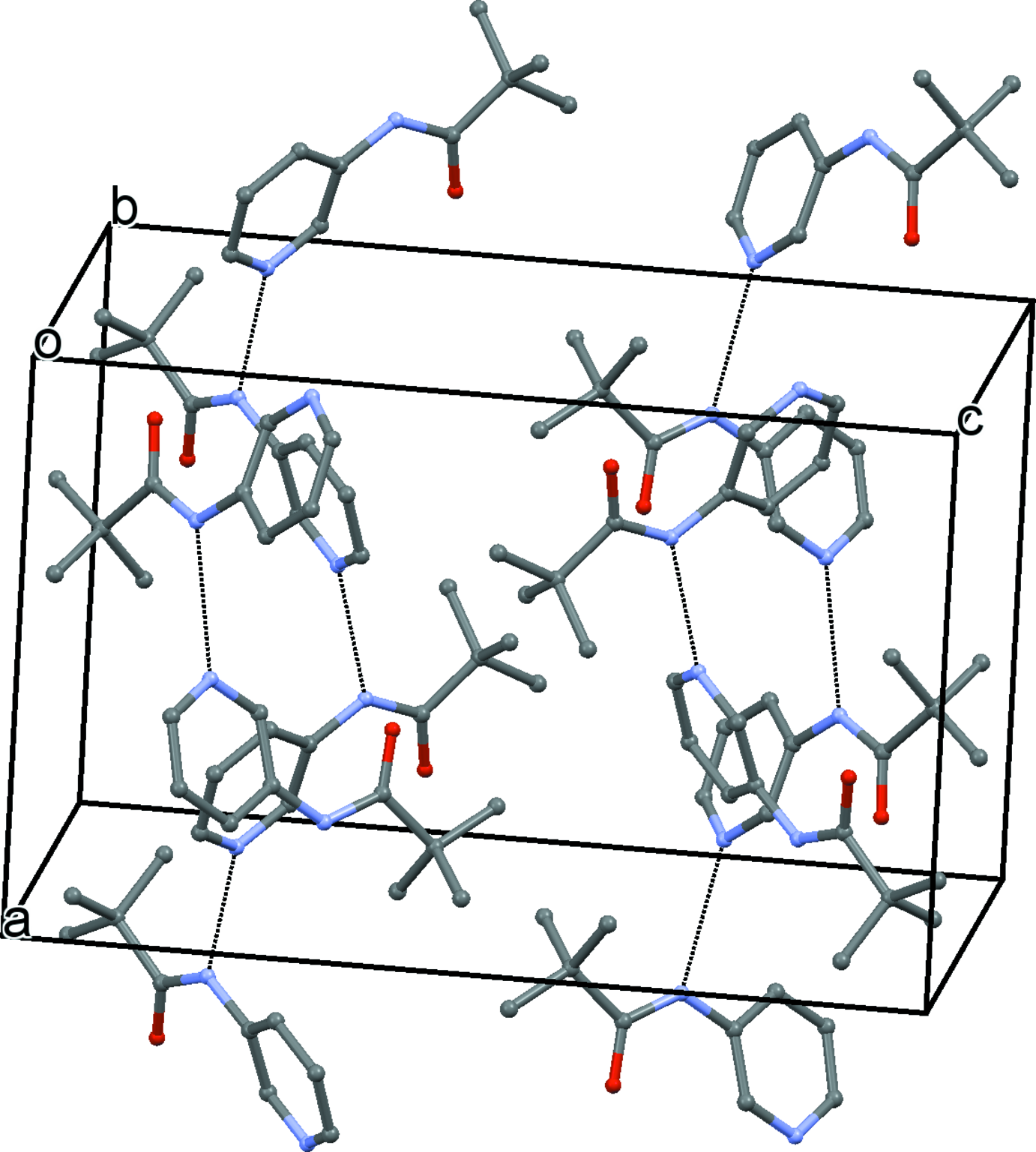

Supplement: Supplementary file 5 [file e-71-0o246-fig2.tif]
